# Impact of vanillin on postharvest disease control of apple

**DOI:** 10.3389/fmicb.2022.979737

**Published:** 2022-08-25

**Authors:** Xiangyu Wang, Xuemin Zhang, Meng Sun, Li Wang, Yaoyuan Zou, Lin Fu, Chuanyu Han, Anqing Li, Limei Li, Chunyu Zhu

**Affiliations:** ^1^College of Life Science, Liaoning University, Shenyang, China; ^2^Jilin Provincial Academy of Forestry Science, Changchun, China

**Keywords:** vanillin, apple, postharvest disease, antifungal mechanism, induced resistance

## Abstract

Apple fruits are susceptible to infection by postharvest fungal pathogens, which may cause fruit decay and severe economic losses. This study investigated the antifungal spectrum of vanillin against common decay pathogens of apple and explored the antifungal mechanisms of vanillin *in vitro*. *In vivo* experiments were carried out to evaluate the effects of vanillin on apple postharvest disease control and fruit quality. Moreover, the induced resistance mechanism of vanillin on apple fruit was preliminarily explored. The results showed that vanillin has broad-spectrum antifungal effects, especially on *Alternaria alternata*. Vanillin could significantly inhibit the growth rate, mycelium biomass, and spore germination of pathogenic fungi by increasing the cell membrane permeability and lipid peroxidation. Importantly, vanillin treatment reduced the incidence of apple decay caused by *A. alternata* and *Penicillium expansum*, and contributed to improve fruit quality. Further studies indicated that vanillin could induce elevation in the activities of defense-related enzymes in apple fruit, such as phenylalanine ammonia-lyase (PAL), chitinase (CHI) and β-1,3-glucanase (β-1,3-GA), and increase total phenols and flavonoids contents. Generally, these results suggest that vanillin may contribute to the induced resistance of apple fruits to pathogenic fungi. To conclude, the results of this research provide theoretical foundations for the application of vanillin in the control of apple postharvest decay caused by fungal pathogens.

## Introduction

Apple (*Malus domestica*) is one of the main dietary fruits for consumption, which has rich nutrition and pleasurable taste (Li et al., 2020a). However, the problem of postharvest decay seriously restricts the economic benefits of apples. Globally, approximately 25 to 50% of apple product losses are caused by fungal pathogens during storage. Pathogenic fungi are the main causes of postharvest decay and quality decline of apple fruits, such as *Alternaria alternata* and *Penicillium expansum* (Zhong et al., 2018). Blue mold caused by *P. expansum* is one of the most common and economically costly postharvest fruit decay diseases in the world. Apple postharvest decay caused by *P. expansum* can cause up to 50% fruit loss, with an estimated annual loss of up to $250 million (Wang et al., 2018; Luciano-Rosario et al., 2020). Black spot rot caused by *A. alternata* is currently an important postharvest disease of apple fruits in China that can be infected and colonized through wounds on the surface of apple fruits during the storage (Shu et al., 2019). These postharvest fungal pathogens not only seriously limit the commercial value of fruits, but also easily produce mycotoxins that are harmful to humans (Wang et al., 2017; Chaudhari et al., 2021). Currently, fungicides are widely used for the prevention of apple postharvest fungal diseases. The persistent application of fungicides such as phenylpyrrole, thiabendazole, fludioxonil and benomyl has great deficiencies (Šernaitė et al., 2020). However, chemical control has great deficiencies. On the one hand, the long-term use of chemical fungicides can develop fungicide resistance in pathogens. On the other hand, chemical fungicides can cause environmental pollution and pose hazards to human health (Deng et al., 2021; Dou et al., 2021). Therefore, it has broad application prospects to develop environmental-friendly, low-cost, and sustainable postharvest disease control methods.

Nowadays, it is considered to be a Generally Recognized As Safe (GRAS) way to preserve fruits with plant extracts or their derivatives (Palou et al., 2016; Loi et al., 2020). These reagents can effectively prevent fruit decay and ensure food safety, which can be used as a new substitute for traditional fungicides (Oulahal and Degraeve, 2022). Chitosan coating can significantly inhibit the growth of *Penicillium citrinum* and *Aspergillus niger*, and enhance the disease resistance of postharvest oranges (Cheng et al., 2019). In addition, chitosan can maintain the balance of reactive oxygen species (ROS) in organisms by increasing the content of antioxidants such as ascorbic acid in the fruit, which is of great significance to prolong the shelf life of raspberry (Tezotto-Uliana et al., 2014). The grapes treated with essential oils have significantly reduced the number of attached microorganisms and the decay rate, which is expected to become a new type of antifungal agent (Pedrotti et al., 2019). Quercetin can inhibit the normal growth and development of pathogenic fungi by destroying the mycelial structure. Furthermore, quercetin can also enhance the activity of antioxidant enzymes in fruit, maintain the balance of the ROS scavenging system and enhance the stress resistance of kiwifruit (Zhang et al., 2018).

Plant phenols are important secondary metabolites in plants with complex structures and multiple functions. Vanillin is a kind of organic phenolic aldehyde compound that widely exists in *Vanilla planifolia*, *Vanilla pompona*, and *Vanilla tahitensis* (Fache et al., 2016). Vanillin has broad-spectrum antimicrobial effects and can be used in foods together with other substances (Li et al., 2020b). Vanillin can be used to control postharvest tomato fruit decay by maintaining constant defense-related enzyme activity such as polyphenol oxidase (PPO), peroxidase (POD), and PAL (Safari et al., 2020, 2021). Vanillin interacts with chitosan to produce Schiff base, which has a significant inhibitory effect on *Escherichia coli* (Stroescu et al., 2015). In addition, chitosan-coated paper containing vanillin can effectively inhibit mango anthracnose and maintain the physicochemical properties (firmness, total soluble solids, titratable acidity, weight, and color) of mango fruit throughout storage (Jaimun and Sangsuwan, 2019). During postharvest storage of grapes, vanillin can effectively resist fruit decay caused by yeast–mold and *Botrytis cinerea*, and maintain higher antioxidant activity and total phenol content of grapes (Takma and Korel, 2017; Sangsuwan and Sutthasupa, 2019).

At present, there are relatively few studies on the application of plant phenols in fruit preservation. As a phenolic aldehyde that can be extracted from plants, vanillin has the characteristics of antimicrobial and environmental protection, which can meet the demand for food safety. This study evaluated the inhibition of vanillin on common pathogens causing apple decay and explored the antifungal mechanisms. In addition, our study also explored the control effect and induced resistance ability of vanillin on apple postharvest diseases. The results are helpful for replacing traditional chemical fungicides with vanillin to control and prevent postharvest diseases of apple fruits.

## Materials and methods

### Materials

Apple fruits (*M. domestica* ‘Hanfu’) with uniform size, no mechanical damage, and consistent maturity were used as experimental materials. The selected apple fruits were surface sterilized by immersing in 2% (v/v) sodium hypochlorite for 2 min, then rinsed with sterile distilled water and naturally dried. Vanillin was purchased from Sigma Co. (Saint Louis, USA), and made into solutions of different concentrations with sterile distilled water. *A. alternata*, *P. expansum*, and *B. cinerea* were identified and preserved in our laboratory. *Neofabraea kienholzii*, *Neofabraea perennans*, *Neofabraea malicorticis*, *Monilinia fructicola*, *Phacidiopycnis washingtonensis*, *Diaporthe perniciosa*, *Sphaeropsis pyriputrescens*, and *Venturia inaequalis* were obtained from Jilin Provincial Academy of Forestry Science (Changchun, China). These pathogenic fungi were maintained on potato dextrose agar (PDA) medium at 28°C. Moreover, the pathogenic fungi were, respectively, made into spore suspensions with sterile distilled water (1 × 10^5^ spores/ml).

### Investigation of antifungal spectrum

The different pathogen spore suspensions (1 × 10^5^ spores/ml) were uniformly coated on PDA medium plates. The sterilized Oxford cup was placed in the center of the plate, and 200 μl of vanillin solution (10.0 mg/ml) was added. An equal volume of sterile distilled water was added to the Oxford cup as a control. The plates were cultured at 28°C, and the diameter of the inhibition zone was recorded. All experiments were performed with three biological replicates.

### *In vitro* antifungal effects of vanillin

*A. alternata* spore suspension (1 × 10^5^ spores/ml) was uniformly coated on the PDA medium plates. The sterilized Oxford cup was placed in the center of the plate, and 200 μl of vanillin solution of different concentrations (0.5, 1.0, 5.0, 10.0, 15.0, and 20.0 mg/ml) was added. An equal volume of sterile distilled water was added to the Oxford cup as a control. The plates were cultured at 28°C, and the diameter of the inhibition zone was recorded.

Preparation of potato dextrose (PD) mediums containing vanillin of different final concentrations (0.5, 1.0, 5.0, 10.0, 15.0, and 20.0 mg/ml), and vanillin free medium as a control. Each group was added to 1 ml of *A. alternata* spore suspension. When the mediums were cultured at 28°C for 1 and 3 days, the mycelia were filtered by a vacuum pump, and weighed after drying.

Each vanillin solution was mixed with *A. alternata* spore suspension in equal volume, and sterile distilled water mixed with the spore suspension was used as a control. After incubation at 28°C for 4, 8, 12, 24, and 48 h, the resulting mixtures were dropped onto the glass slide and the germination rate of spores in each group was counted under a microscope.

All experiments were performed with three biological replicates.

### Effects of vanillin on physiology of *Alternaria alternata*

The wet mycelium (0.5 g) was taken out from the mixture and added to the sterile centrifuge tube together with 5 ml of vanillin solution (10 mg/ml), and the control group was treated with an equal volume of sterile water. Then the malondialdehyde (MDA) content of the mixture was determined at 4, 8, 12, 24, and 48 h. The MDA content was determined according to the method of Gong et al. (2021).

Ten mycelial plugs (5 mm in diameter) were added to PD medium and incubated on a shaker for 5 days (150 rpm, 28°C). The incubated mixture was used to measure conductivity and MDA content. The vanillin solution with the final concentration of 10 mg/ml was added in the incubated mixture, and an equal volume of sterile distilled water was added in the incubated mixture as a control. Then the conductivity of the mixture was measured at 4, 8, 12, 24, and 48 h.

All experiments were performed with three biological replicates.

### Vanillin inhibition of postharvest fungal decay in apple

The apple fruits were prepared for inoculation by creating one wound (3 mm depth × 3 mm diameter) on the opposite sides of the fruit equator using a sterile pipette gun head. Each wound was injected with 20 μl of various concentrations (0.5, 1.0, 2.0, 3.0, 4.0, 5.0, and 6.0 mg/ml) of vanillin solution, and sterile distilled water was used as a control. The treated apples were placed at 25°C and 90% relative humidity (RH) for 24 h. Then, 30 μl of *A. alternata* or *P. expansum* spore suspension was reinjected. At 5 days post-inoculation, the incidence rate and lesion diameter of the treatment group and the control group were determined. The incidence rate was calculated as follows: number of decayed wounds/total number of wounds × 100. All experiments were performed with three biological replicates.

### Evaluation of fruit quality

Apple fruits were soaked in vanillin solution (2.0 mg/ml) for 20 min, and sterile distilled water was used as a control. Firmness and soluble solids of fruits were determined at time 0, 1, 2, 3, 4, and 5 days of storage at 25°C and 90% RH. The firmness was determined with a Fruit Texture Analyzer (Stable Micro Systems, United Kingdom) using a diameter probe (Guerra et al., 2015). The total soluble solids content was measured according to the method of Safari et al. (2021). All experiments were performed with three biological replicates (each replicate contained six apples).

### Determination of defense-related enzymes activities

Apple fruits were soaked in vanillin solution (2.0 mg/ml) for 20 min, and sterile distilled water was used as a control. The enzyme activities of PAL, CHI, and β-1,3-GA in fruit samples were determined at time 0, 1, 2, 3, 4, and 5 days of storage at 25°C and 90% RH. Crude enzyme solution was extracted according to the method described by Pan et al. (2020). The enzyme activity of PAL was determined using the methods of Wang et al. (2019b) and Kou et al. (2021) with some modifications. One milliliter of crude enzyme was added into 3 ml of 50 mm borate buffer with 10 mm phenylalanine. The absorbance at 290 nm was measured, and 1 ml of 50 mm PBS was used as a control. An increase of 0.01 absorbance units per hour at 290 nm was taken as one PAL unit, and the activity was expressed as U/g FW (fresh weight). The enzyme activity of CHI was measured using the method of Zheng et al. (2011). The reaction mixture contained 0.5 ml of crude enzyme solution, 0.5 ml of colloidal chitin solution, and 0.5 ml of acetic acid–sodium acetate buffer. After mixture was in 37°C water bath for 1 h, 0.1 ml of desalted snail enzyme was added in mixture, and then heated at 37°C for 1 h. The mixture was added with 0.4 ml of 0.6 mol/l potassium tetraborate and heated at 37°C for 5 min to terminate the reaction. Finally, 1 ml of 100 g/l 4-dimethylaminobenzaldehyde was added to the mixture, and the absorbance at 585 nm was measured. One CHI unit was defined as 1 μg of N-acetylglucosamine from colloidal chitin produced per hour at 585 nm, and the activity was expressed as U/g FW. β-1,3-GA activity was assayed as described by Zong et al. (2010). The reaction system comprised 0.03 ml of crude enzyme solution and 0.07 ml of laminarin and incubated at 37°C for 30 min. The mixture was added with 1.5 ml of 3,5-dinitrosalicylic acid, and in a water bath at 100°C for 5 min to terminate the reaction. The absorbance was measured at 540 nm, one β-1,3-GA unit was defined as the decomposition of laminarin per hour producing 1 mg of glucose. All experiments were performed with three biological replicates (each replicate contained six apples).

### Determination of total phenols content and flavonoids content

Apple fruits were soaked in vanillin solution (2.0 mg/ml) for 20 min, and sterile distilled water was used as a control. Fruit samples were used to determine the contents of total phenols and flavonoids at time 0, 1, 2, 3, 4, and 5 days of storage at 25°C and 90% RH. The contents of total phenols and flavonoids were determined as described by Chen et al. (2015) and Zheng et al. (2021) with appropriate modifications. A total of 3 g of sample was homogenized in 10 ml of 0.5% acetic acid and 70% acetone, then incubated in complete darkness for 24 h. The homogenates were centrifuged at 10,000 × g for 20 min at 4°C. To measure total phenols content, 2 ml of Folin–Ciocalteu reagent and 2 ml of 7.5% sodium carbonate were added to 1 ml of extracting solution. The absorbance at 760 nm was measured, and the results were analyzed by gallic acid standard curve. To measure flavonoid content, 3 ml of extracting solution was mixed with 0.5 ml of 10% aluminum chloride hexahydrate and 0.5 ml of 5% sodium nitrite and allowed to stand for 5 min. Then, the solution was mixed with 1 ml of NaOH (1 mol/l). The absorbance at 510 nm was measured, and the results were analyzed by a catechin standard curve. All experiments were performed with three biological replicates (each replicate contained six apples).

### Statistical analysis

All data of experiments were the mean ± standard deviation (SD) of three biological replicates. Statistical analysis was performed by one-way ANOVA using the IBM SPSS Statistics 22 software (SPSS Inc., Chicago, IL, United States). Duncan’s multiple range test was used to determine the significant differences (*p* < 0.05 or *p* < 0.01).

## Results

### Inhibition spectrum of vanillin against various pathogenic fungi

As shown in Table 1, vanillin solution had a certain inhibitory effect on all test fungi. Vanillin had the strongest inhibitory effect on *A. alternata*, with an obvious inhibition zone of 28.39 mm in diameter. Vanillin also had a strong inhibition on *B. cinerea*, *N. kienholzii*, *N. perennans*, *N. malacorticis*, and *M. fructicola*, and the diameters of these inhibition zones were greater than 20 mm. However, the inhibition effects were relatively weak on *D. perniciosa*, *S. pyriputrescens* and *V. inaequalis*. Due to vanillin having the strongest inhibitory effect on *A. alternata*, it was selected for the follow-up study.

**Table 1:** Antifungal spectrum of vanillin.

**Pathogens**	**Diameter of inhibition zone (mm)**
*A. alternata*	28.39 ± 0.83 a
*P. expansum*	18.71 ± 0.59 d
*B. cinerea*	26.89 ± 0.21 a
*N. kienholzii*	23.59 ± 0.41 b
*N. perennans*	23.08 ± 1.65 b
*N. malacorticis*	20.97 ± 0.97 c
*M. fructicola*	22.54 ± 0.56 bc
*P. washingtonensis*	16.31 ± 0.30 e
*D. perniciosa*	10.15 ± 0.54 g
*S. pyriputrescens*	8.32 ± 0.61 h
*V. inaequalis*	7.31 ± 0.18 h

Significance is represented in alphabetical notation (*p* < 0.05).

### Effects of vanillin on the growth of *Alternaria alternata*

**Figure 1:**
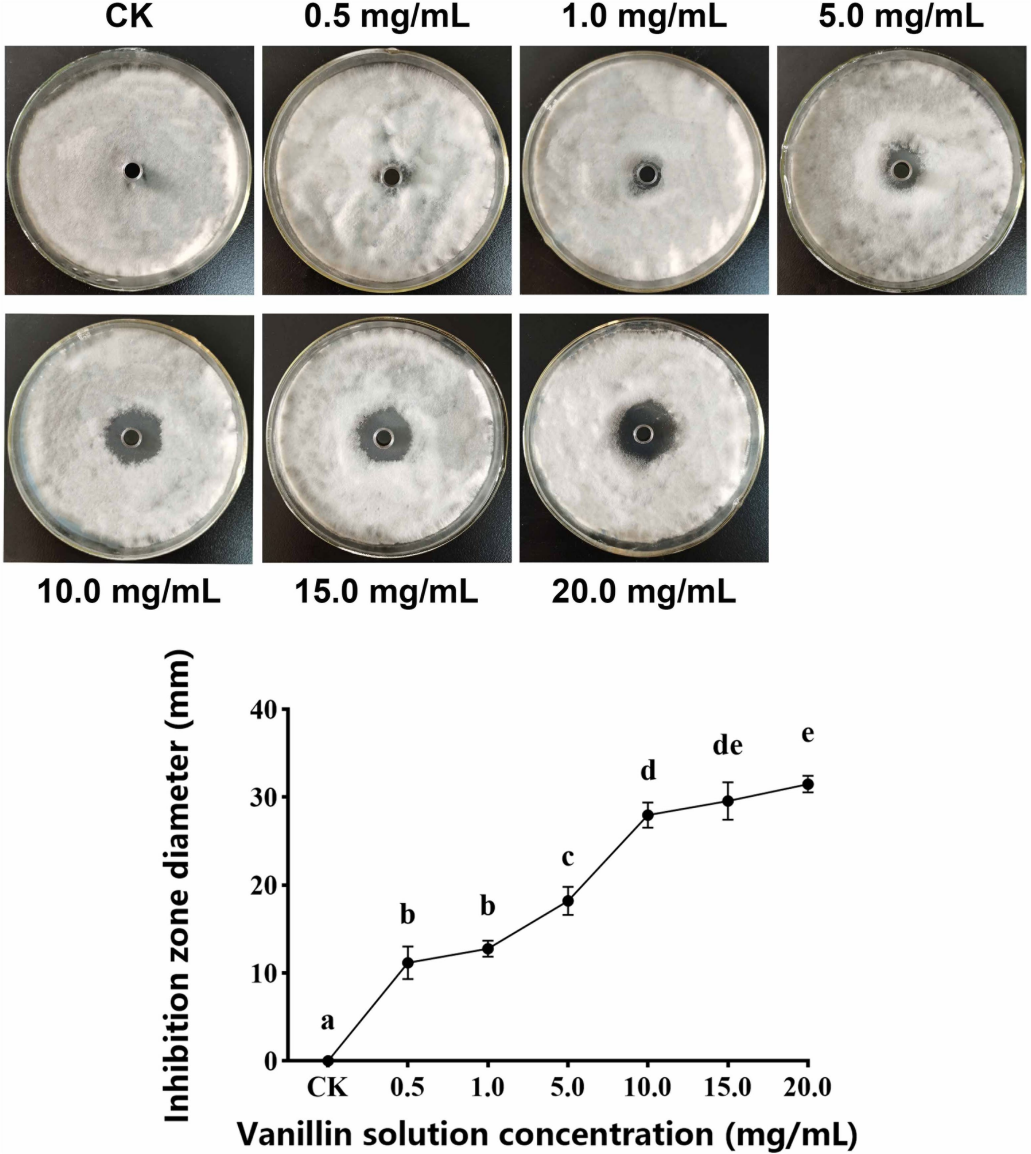
Effects of different concentrations of vanillin on plate assay. Significance is represented in alphabetical notation (*p* < 0.05).

**Figure 2:**
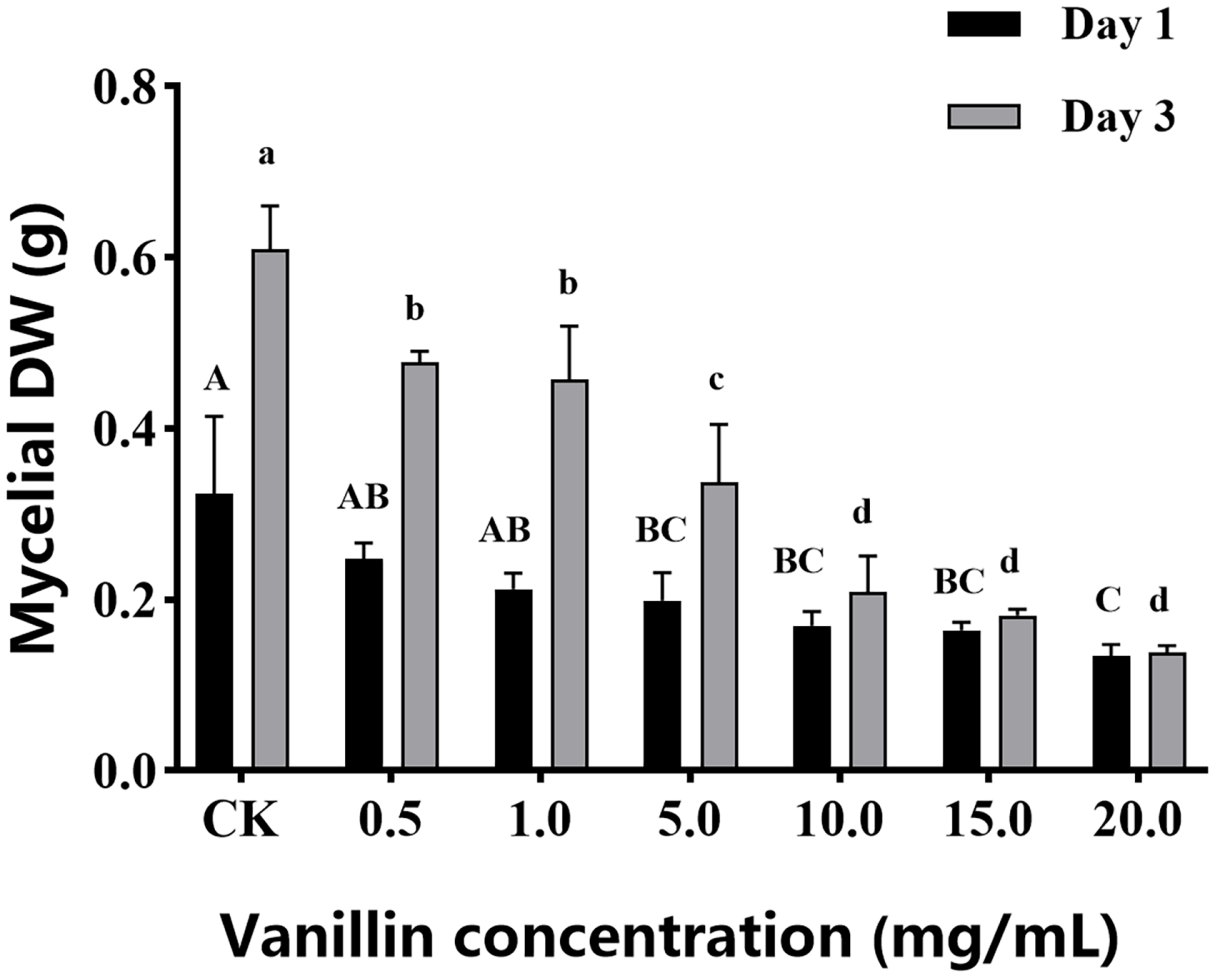
Different concentrations of vanillin on mycelial DW of *Alternaria alternata* control. Significance is represented in alphabetical notation (*p* < 0.05).

**Figure 3:**
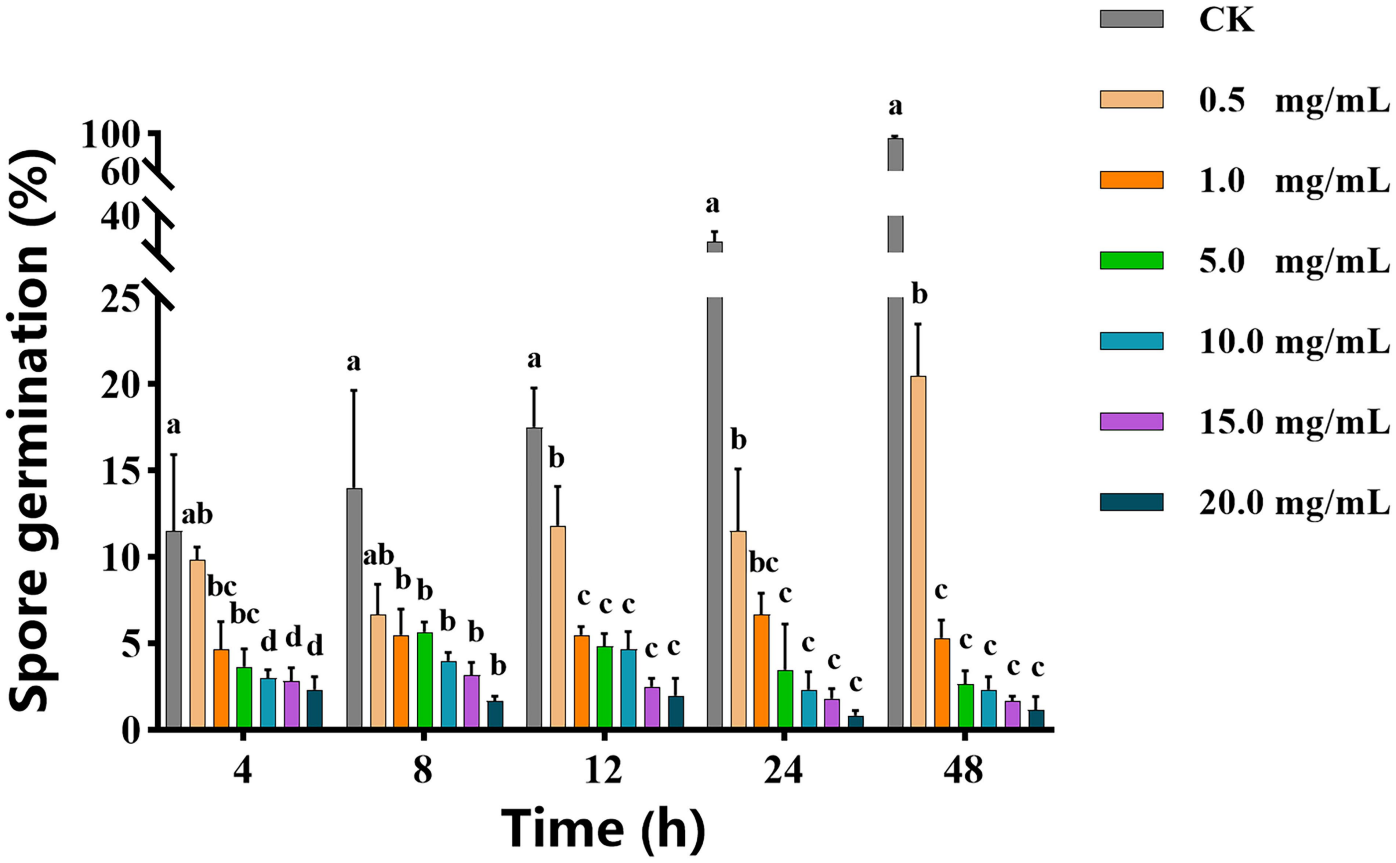
Effects of vanillin on spore germination of *Alternaria alternata*. Significance is represented in alphabetical notation (*p* < 0.05).

As shown in Figure 1, with increasing of vanillin solution concentration, the diameter of the inhibition zone against *A. alternata* gradually expanded. When the concentration of vanillin solution was 10.0 mg/ml, the inhibition zone was significantly improved. However, compared with 15.0 mg/ml, there was no significant difference. As shown in Figure 2, with the increase of time, the dry weight (DW) of mycelium at 3 days was significantly higher than that at 1 day, indicating that the growth of *A. alternata* was in good condition. When treated with vanillin solution, the DW of mycelium in the treated group decreased and its normal growth was inhibited. As the concentration of vanillin solution increased, the DW of mycelium showed a downward trend. At 1 day for 5.0 mg/ml vanillin solution treatment, the DW of mycelium in the treatment group was approximately 2/3 of that in the control group. At 3 days, vanillin concentration of 10.0 mg/ml and above had obvious inhibition effects, and the DW of mycelia in the treatment group was significantly higher than that in the control group. Figure 3 shows that the spore germination rate of the control group increased obviously from 12 h and reached more than 90% at 48 h. In contrast, the spore germination rate of *A. alternata* decreased after vanillin treatment, and vanillin at a concentration of 1 mg/ml could significantly inhibit the spore germination of *A. alternata*. At 4 h of treatment, when the vanillin concentration was 10.0 mg/ml, the inhibition effect was significantly higher than that at 5.0 mg/ml, and the germination rate decreased to 3%. It can be seen that vanillin solution has an inhibitory effect on spore germination, and the inhibitory effect is affected by concentration and treatment time. The results indicated that the vanillin solution at 10.0 mg/ml was suitable for the next study.

### Inhibitory effect of vanillin on physiology of *Alternaria alternata*

**Figure 4:**
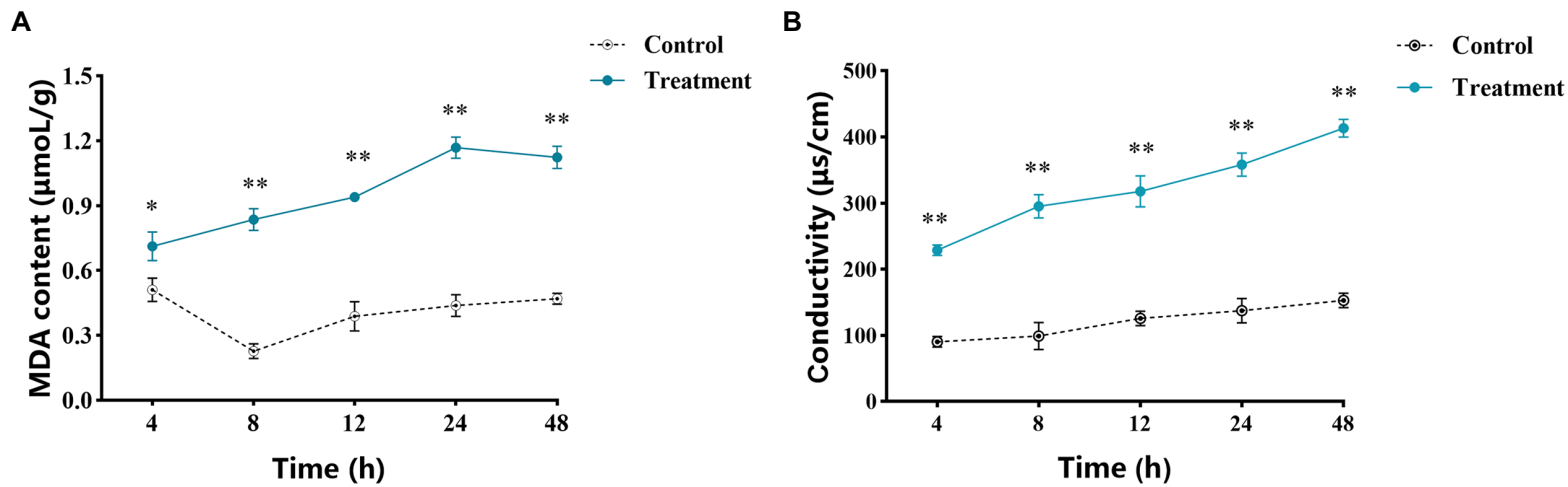
Effects of vanillin on the cell membrane of *Alternaria alternata*. **(A)** MDA content of *A. alternata*. **(B)** Extracellular conductivity of *A. alternata*. Significance is represented by ^*^p < 0.05 and ^**^p < 0.01.

The determination results of MDA content are shown in Figure 4A. The MDA content of the treatment group was always higher than that of the control group. The MDA content in the treatment group reached the peak at 24 h, which was 2.66 times that of the control group. Moreover, compared with the control, the MDA content of *A. alternata* was significantly increased after 10.0 mg/ml vanillin treatment.

By measuring the extracellular conductivity of *A. alternata*, it was found that the conductivity of the control group and the treatment group increased with the extension of time (Figure 4B). However, compared with the control group, the conductivity of the treatment group increased higher. At 48 h, the conductivity of the treatment group reached the highest value, which was 2.7 fold that of the control group, indicating that the cell membrane was most severely damaged at this time. In the whole process, the extracellular conductivity of *A. alternata* treated with vanillin solution was significantly higher than that of the control group, and showed a continuous increasing trend, indicating that the electrolyte in the mycelia treated with vanillin solution was extravasated, the permeability of the cell membrane was enhanced, and the integrity of the cell membrane was destroyed.

### Inhibitory effect of vanillin against postharvest decay caused by *Alternaria alternata* and *Penicillium expansum*

**Figure 5:**
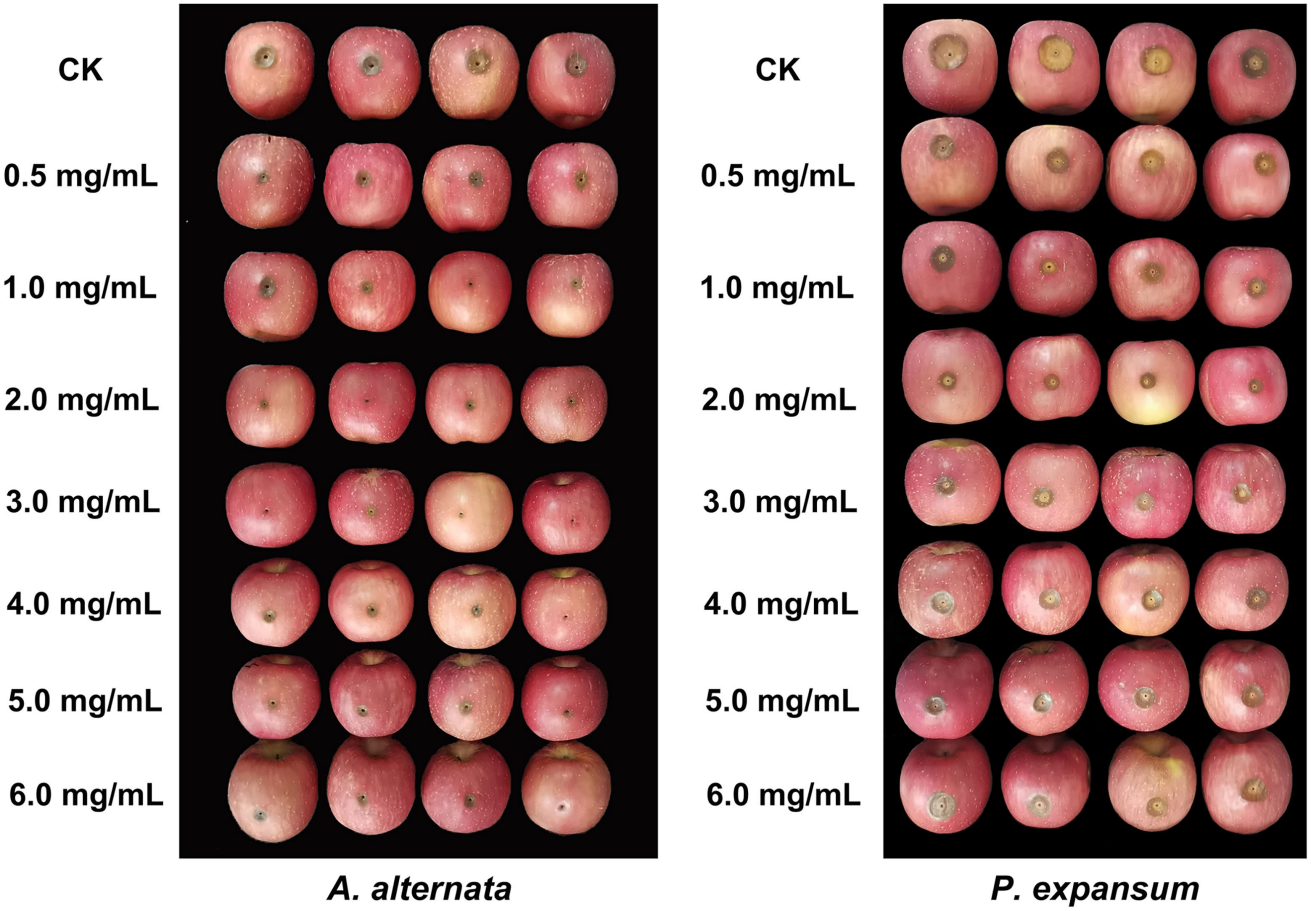
Effects of different concentrations of vanillin on postharvest decay in apples caused by *Alternaria alternata* and *Penicillium expansum*.

The effect of vanillin treatment was investigated on apples inoculated with two postharvest pathogens (Figure 5; Table 2). Treatment with vanillin significantly reduced the lesion diameter of apple fruits infected with pathogenic fungi. As shown in the results, the inhibitory effect of vanillin on *A. alternata* and *P. expansum* infection of apples varied with the concentration of vanillin. According to the comprehensive data, the lesion diameter decreased significantly after treatment with 2.0 mg/ml vanillin solution. After that, the vanillin concentration continued to increase, whereas the effect was not obvious. Therefore, 2.0 mg/ml was selected as the most appropriate concentration for follow-up studies.

**Table 2:** Different concentrations of vanillin on postharvest decay control.

**Vanillin solution concentration**	**Lesion diameter caused by *A. alternata* (mm)**	**Lesion diameter caused by *P. expansum* (mm)**
0	17.46 ± 0.31 a	21.29 ± 0.63 a
0.5	12.97 ± 0.62 b	15.56 ± 0.60 b
1.0	13.49 ± 0.81 b	14.05 ± 0.59 cd
2.0	8.42 ± 0.88 e	10.81 ± 0.32 f
3.0	8.69 ± 0.43 e	12.73 ± 1.17 e
4.0	9.01 ± 1.29 de	13.24 ± 0.37 de
5.0	10.33 ± 0.30 cd	12.29 ± 0.33 e
6.0	11.10 ± 1.10 c	14.70 ± 0.96 bc

Significance is represented in alphabetical notation (*p* < 0.05).

### Effects of vanillin on apple fruit quality

**Figure 6:**
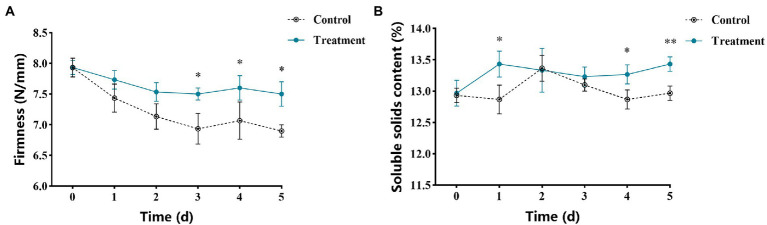
Effects of vanillin treatment on fruit quality. **(A)** Firmness of apple fruit. **(B)** Soluble solids content of apple fruit. Significance is represented by ^*^p < 0.05 and ^**^p < 0.01.

The changes of firmness of apple fruits after vanillin treatment are shown in Figure 6A. The results showed that the effect of storage time on fruit firmness showed a downward trend. After 3 days of storage, the fruit firmness of the vanillin-treated group was significantly higher than that of the control group. Therefore, vanillin treatment could promote an increase in fruit firmness during storage. Figure 6B shows the change of soluble solids content in apple fruit after vanillin treatment. The soluble solids content of the control group peaked on the second day of storage. Nevertheless, compared with the control group, the soluble solids content of vanillin-treated group was significantly higher at 1 and 4 days. As the results showed, vanillin treatment could also increase soluble solids content in apple fruit.

### Effects of vanillin on activities of defense-related enzymes in apple

**Figure 7:**
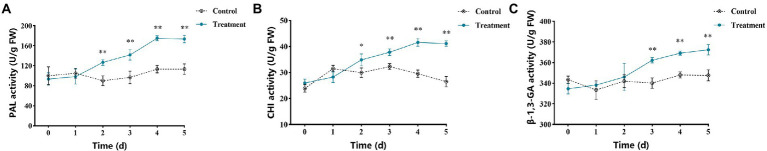
Effects of vanillin treatment on the activities of PAL **(A)**, CHI **(B)**, and β-1,3-GA **(C)** in apple fruit. Significance is represented by ^*^p < 0.05 and ^**^p < 0.01.

Figure 7A shows the changes of PAL enzyme activity in apple fruits after vanillin treatment. The results showed that the change of PAL enzyme activity in the control group was not obvious. Compared with the control group, the PAL enzyme activity of the vanillin-treated group showed a significant difference and increasing trend at 2 days and reached the peak at 4 days. CHI activity results are shown in Figure 7B. At 1 day, there was little difference between the control and treatment groups. However, compared with the control group, the CHI activity of the vanillin-treated group had a significant difference and showed an upward trend after 2 days of storage. As shown in Figure 7C, vanillin treatment had a significant effect on inducing β-1,3-GA activity during apple fruit storage. β-1,3-GA activity of the vanillin-treated group increased significantly after 3 days, whereas the change of enzyme activity in the control group was not significant.

### Effects of vanillin on fruit total phenols content and flavonoids content

**Figure 8:**
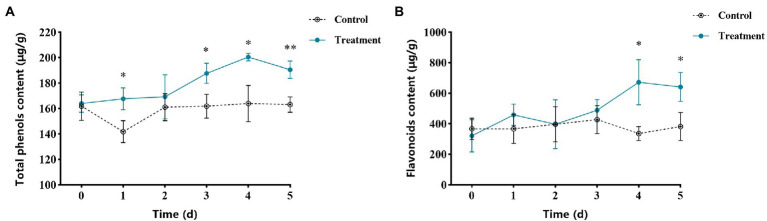
Effects of vanillin treatment on the contents of total phenols **(A)** and flavonoids **(B)** in apple fruit. Significance is represented by ^*^p < 0.05 and ^**^p < 0.01.

The total phenols content of apple fruits treated with vanillin is shown in Figure 8A. The total phenols content in the control group was almost unchanged, while after vanillin treatment, the total phenols content increased significantly. Fruits treated with vanillin showed the highest total phenols content during storage. Obviously, the highest total phenol content was at 4 days post treatment, which was more than 1.25 fold than the control group. The flavonoid content at different times during storage is shown in Figure 8B. The total phenols content of the control group had little change during storage. Compared with the control group, the flavonoid content of vanillin-treated group reached the peak at 4 days, which was significantly higher than that of the control group.

## Discussion

Apple fruits are often infested by pathogenic fungi during postharvest storage, which results in huge economic losses annually (Zhang et al., 2017a). Chemical fungicides have always been the main method to control postharvest diseases (Lahlali et al., 2020). Due to the resistance of pathogens to fungicides and safety considerations, compounds extracted from plants are increasingly used for postharvest fruit protection (Šernaitė et al., 2020). Vanillin can be used as a safe and non-toxic natural food preservative that is widely used in fruits and vegetables, but its ability to inhibit fruit postharvest disease has rarely been tested. Specifically, studies on the inhibitory effect of vanillin on postharvest disease in apples have not been conducted.

Our results showed that vanillin could significantly inhibit the growth of pathogenic fungi causing apple postharvest decay *in vitro* and *in vivo*. The present study revealed that vanillin can inhibit the growth of most apple decay fungi and has broad-spectrum antifungal effects, among which the inhibitory effect on *A. alternata* was the most significant. In our study, the mycelial DW and spore germination rate of *A. alternata* treated with vanillin decreased with increasing of vanillin concentration. These results are similar to the previous report that vanillin can inhibit the spore germination of *Aspergillus flavus* (Li et al., 2021). In view of the practical application in fruits, the inhibition of mycelial growth and spore germination by vanillin is noteworthy because spores are important structures for the spread and survival of pathogenic fungi, resulting in fruit infection and decay (de Souza et al., 2015). Vanillin at a concentration of 10.0 mg/ml provided significant inhibition against pathogenic fungus *in vitro*. However, vanillin above this concentration showed relatively little improvement in inhibiting fungal pathogens. At the same time, we determined 10.0 mg/ml to be the optimal inhibitory concentration of vanillin for subsequent *in vitro* experiments. A complete cell membrane system can ensure the basic physiological activities of cells. Since the plasma membrane is an important target of fungicides, we investigated whether vanillin interacts with the cell membrane of pathogenic fungi. Furthermore, the disruption of membrane integrity due to the oxidation of membrane lipids by ROS is part of effective antifungal action modes (Rautenbach et al., 2016). Increased MDA leads to membrane damage and electrolyte leakage, reduces the fluidity of the cell membrane, significantly increases permeability, and finally leads to changes in cell structure and function (Abdelhai et al., 2019; Qu et al., 2020). Excessive ROS can attack unsaturated fatty acids in lipids, resulting in lipid peroxidation. Our results showed that vanillin solution could cause membrane lipid peroxidation of mycelium, resulting in an increase in the content of MDA, which had an impact on the cell structure and function. The MDA content in the vanillin-treated group increased significantly, suggesting that vanillin induced severe lipid peroxidation and produced excessive oxidative damage to the cell membrane of pathogenic fungi. Correspondingly, the conductivity of the treatment group was significantly higher than that of the control group. The results indicated that cell membrane permeability of *A. alternata* was damaged so that contents such as electrolytes were leaked out. Our results suggest that vanillin exerts antifungal activity directly *via* membrane damage and oxidative stress against pathogenic fungi, which ultimately leads to the inhibition of mycelial growth and spore germination.

Currently, *A. alternata* and *P. expansum* are the main pathogenic fungi responsible for severe postharvest losses in apples. In our study, *A. alternata* and *P. expansum* were used as pathogens to explore the control effect of vanillin on apple postharvest decay. As can be seen from the results, vanillin treatment significantly reduced the lesion diameters and in apple fruits inoculated with *A. alternata* and *P. expansum* (Figure 5; Table 2). Our comprehensive analysis determined that the vanillin solution with a concentration of 2.0 mg/ml can effectively protect against postharvest decay in apples. Concentrations above or below 2.0 mg/ml were comparatively less efficient in inhibiting postharvest decay. As one of the indexes of fruit quality, firmness is also an important factor for fruit to resist the invasion of pathogenic fungi (Pan et al., 2021). Fruits are prone to soften during storage or attacked by pathogens (Barreto et al., 2016). It is remarkable that compared with the control group, apple fruits treated with vanillin showed greater firmness values during storage (Figure 6A). The results showed that vanillin could effectively delay the tendency of fruit firmness to decrease. These results demonstrate that the antifungal effects of vanillin treatment may also contribute to the maintenance of firmness because it can resist the infection of fungal cell wall degrading enzymes. Soluble solids are consumed by respiration to support the normal activities of life during storage, and soluble solids content is another indicator to evaluate fruit quality (Gao et al., 2013; Zhang et al., 2017b). The results in Figure 6B indicate that there was a significant difference between the vanillin-treated group and the control group. Compared to the control group, treatment with vanillin significantly reduced the loss of soluble solids. Therefore, vanillin showed the ability to inhibit apple postharvest decay and improve fruit quality.

Compared to the antifungal activity *in vitro*, a lower concentration of vanillin was needed to effectively inhibit apple fruit postharvest decay. This may be since vanillin can also induce resistance responses in apple tissues. PAL, CHI and β-1,3-GA, are important enzymes that contribute to innate disease resistance in plants. The activity levels of these enzymes are positively correlated with fruit disease resistance and could be used as indicators for inducing fruit disease resistance levels. PAL is a crucial enzyme in the phenylpropanoid metabolic pathway, which is essential for normal growth, development and resistance of plants (Qin et al., 2015; Wang et al., 2019a). In plant disease resistance, PAL can promote the production of secondary metabolites, including total phenols, flavonoids and lignin (Romanazzi et al., 2016; Wang et al., 2016; Ge et al., 2021). The contents of total phenols and flavonoids were positively correlated with plant disease resistance (Zhao et al., 2021). In the process of plant disease resistance, different elicitors can effectively induce the accumulation of total phenols content. Total phenols are major secondary metabolites, which are closely related to the mechanism of plant disease resistance. Phenolic compounds play a vital role in improving the host defense system against pathogens as components of cell walls (Champa et al., 2015). Flavonoids are secondary metabolites of polyphenols, which play the roles of phytoanticipins and phytoalexins in plant defense against pathogens (Desta et al., 2016). PAL can catalyze phenylalanine to cinnamoyl-CoA, which is the precursor of the flavonoid metabolic pathway (Deng and Lu, 2017). Our study investigated that the treatment of apple fruits with vanillin increased PAL activity. This suggests that vanillin may modulate the phenylpropanoid metabolic pathway in apples. Consistent with this view, vanillin treatment could also increase the contents of total phenols and flavonoids in apple fruits (Figure 8). Therefore, vanillin can be used as an elicitor to activate the phenylpropanoid metabolic pathway in apple defense system.

Pathogenesis-related proteins (PRs) are a class of proteins produced by plants under the stimulation of external stress conditions, which are closely related to plant disease resistance. CHI and β-1,3-GA are the main PR members and are considered as marker enzymes involved in plant systemic resistance (Wojtasik et al., 2019). CHI has direct microbial inhibition, which can degrade the cell wall of most pathogens. β-1,3-GA can restrict the growth of mycelium by targeting β-1,3-glucan on the pathogenic cell wall (Pan et al., 2020). The results of this experiment showed that vanillin treatment could significantly enhance the enzyme activities of CHI and β-1,3-GA in fruits (Figure 7), which is contributed to enhance the resistance to pathogens. In present study, PAL, CHI and β-1,3-GA activity significantly increased in apple fruits treated with vanillin, suggesting that vanillin can induce fruit resistance to resist the invasion of pathogens. Furthermore, the increased activity of defense related enzymes enhances resistance by promoting the synthesis of antifungal compounds in apples.

## Conclusion

This study investigated the effect of vanillin in inhibiting postharvest decay of apple fruit. Vanillin can significantly inhibit the growth of postharvest pathogenic fungi, and the antifungal effect is attributed to the destruction of cell membrane integrity. The results of the present study demonstrated that vanillin could effectively reduce the symptoms of apple postharvest decay caused by *A. alternata* and *P. expansum*. Furthermore, vanillin has the ability to induce the up-regulation of defense-related enzyme activity of PAL, CHI and β-1,3-GA in apple fruits. Meanwhile, vanillin could also increase the contents of total phenols and flavonoids in fruits. Therefore, the disease resistance induced by vanillin was associated with the positive regulation of the phenylpropane pathway. The above findings suggest that vanillin is a feasible treatment for apple fruit during postharvest storage.

## Data availability statement

The raw data supporting the conclusions of this article will be made available by the authors, without undue reservation.

## Author contributions

XW, LL, and CZ conceived and designed the research. XZ performed the experiments. XW performed the data analyses and wrote the paper. LF and CH contributed to analysis of data. MS, LW, YZ, and AL provided experiment assistance. All authors contributed to the article and approved the submitted version.

## Funding

The research was supported by People’s Livelihood Science and Technology Program of Liaoning Provincial Department of Science and Technology (2021JH2/10200015), Youth Fund of Liaoning Provincial Education Department (No. LQN202001), General Project of Liaoning Provincial Education Department (No. LJC201910), National Natural Science Foundation of China (No. 41907282), and Youth Scientific Research Fund of Liaoning University (No. LDQN-202010).

## Conflict of interest

The authors declare that the research was conducted in the absence of any commercial or financial relationships that could be construed as a potential conflict of interest.

## Publisher’s note

All claims expressed in this article are solely those of the authors and do not necessarily represent those of their affiliated organizations, or those of the publisher, the editors and the reviewers. Any product that may be evaluated in this article, or claim that may be made by its manufacturer, is not guaranteed or endorsed by the publisher.
